# Opposite environmental and genetic influences on body size in North American *Drosophila pseudoobscura*

**DOI:** 10.1186/s12862-015-0323-3

**Published:** 2015-03-21

**Authors:** Michelle L Taylor, Alison Skeats, Alastair J Wilson, Tom A R Price, Nina Wedell

**Affiliations:** College of Life and Environmental Sciences, Biosciences, University of Exeter, Penryn Campus, Penryn, Cornwall TR10 9FE UK; Institute of Integrative Biology, University of Liverpool, Crown Street, Liverpool, L69 7ZB UK

**Keywords:** Temperature-size rule, Bergmann clines, Local selection, Phenotypic plasticity, Selection, *Drosophila pseudoobscura*

## Abstract

**Background:**

Populations of a species often differ in key traits. However, it is rarely known whether these differences are associated with genetic variation and evolved differences between populations, or are instead simply a plastic response to environmental differences experienced by the populations. Here we examine the interplay of plasticity and direct genetic control by investigating temperature-size relationships in populations of *Drosophila pseudoobscura* from North America. We used 27 isolines from three populations and exposed them to four temperature regimes (16°C, 20°C, 23°C, 26°C) to examine environmental, genetic and genotype-by-environment sources of variance in wing size.

**Results:**

By far the largest contribution to variation in wing size came from rearing temperature, with the largest flies emerging from the coolest temperatures. However, we also found a genetic signature that was counter to this pattern as flies originating from the northern, cooler population were consistently smaller than conspecifics from more southern, warmer populations when reared under the same laboratory conditions.

**Conclusions:**

We conclude that local selection on body size appears to be acting counter to the environmental effect of temperature. We find no evidence that local adaptation in phenotypic plasticity can explain this result, and suggest indirect selection on traits closely linked with body size, or patterns of chromosome inversion may instead be driving this relationship.

**Electronic supplementary material:**

The online version of this article (doi:10.1186/s12862-015-0323-3) contains supplementary material, which is available to authorized users.

## Background

Recent work has highlighted the role of phenotypic plasticity in evolutionary divergence [1-4]. The ability to express alternative phenotypic characters in response to a range of environmental stimuli such as predators, rivals, or abiotic environmental properties can both expose and protect genetic variation across environments, effectively strengthening or diluting selection on individual traits [4-7]. By studying the phenotypic plasticity of traits expressed by individual genotypes across a range of environments, we can determine both the extent of trait plasticity, the genetic variation supporting it and the constraints that arise through specific genotype-by-environment interactions (GxEs) [8,9]. This can indicate the driving factors behind patterns of phenotypic variation across environments and help to explain within-species patterns of divergence in morphological, behavioural or physiological traits [4,10,11].

The well-known relationship between temperature and body size, known as the temperature-size rule, provides a useful framework for examining phenotypic plasticity in nature [12-15]. This is because body sizes produced from phenotypically plastic responses to environmental temperatures can become genetically fixed over time [3]. Individuals from populations experiencing different climates then demonstrate fixed differences in body sizes, even under standard laboratory conditions [16-19]. Temperature-size relationships that exist across latitudinal or altitudinal gradients are known as Bergmann clines and are typically characterized by larger body sizes at cooler temperatures, i.e. higher latitudes or altitudes [20,21]. However, the *converse* Bergmann cline, where individuals in cooler climates (high latitudes or altitudes) mature at *smaller* sizes has also been observed [20]. Converse Bergmann clines may be adaptive, for example to accommodate a shorter growing season than that available to tropical conspecifics, or represent a conflict between developmentally plastic responses to temperature and adaptive responses to seasonal variation [16]. Opposing environmental and genetic influences on phenotypic traits, known as countergradient variation, may result in very little observable phenotypic variation across the geographic range of a species [22]. Direct conflicts between environmental effects and adaptive selection on influential traits such as body size may lead to correlated traits being constrained or even maladapted in different populations of species occupying large geographic ranges [22]. For example, female body size can be directly linked to fecundity and survival and play a large role in the success of populations occupying challenging environments, such as northern populations that endure long overwintering periods [23]. Body size can also influence the mating success of males, which may impact on the genetic variation in populations of different densities [24]. Body size can also affect the ability to tolerate desiccation and cold-stress which may ultimately determine species distribution patterns [25,26]. Temperature-size relationships and Bergmann clines in widely distributed species therefore represent an ideal framework within which to examine the evolutionary role of phenotypic plasticity.

**Figure 1:**
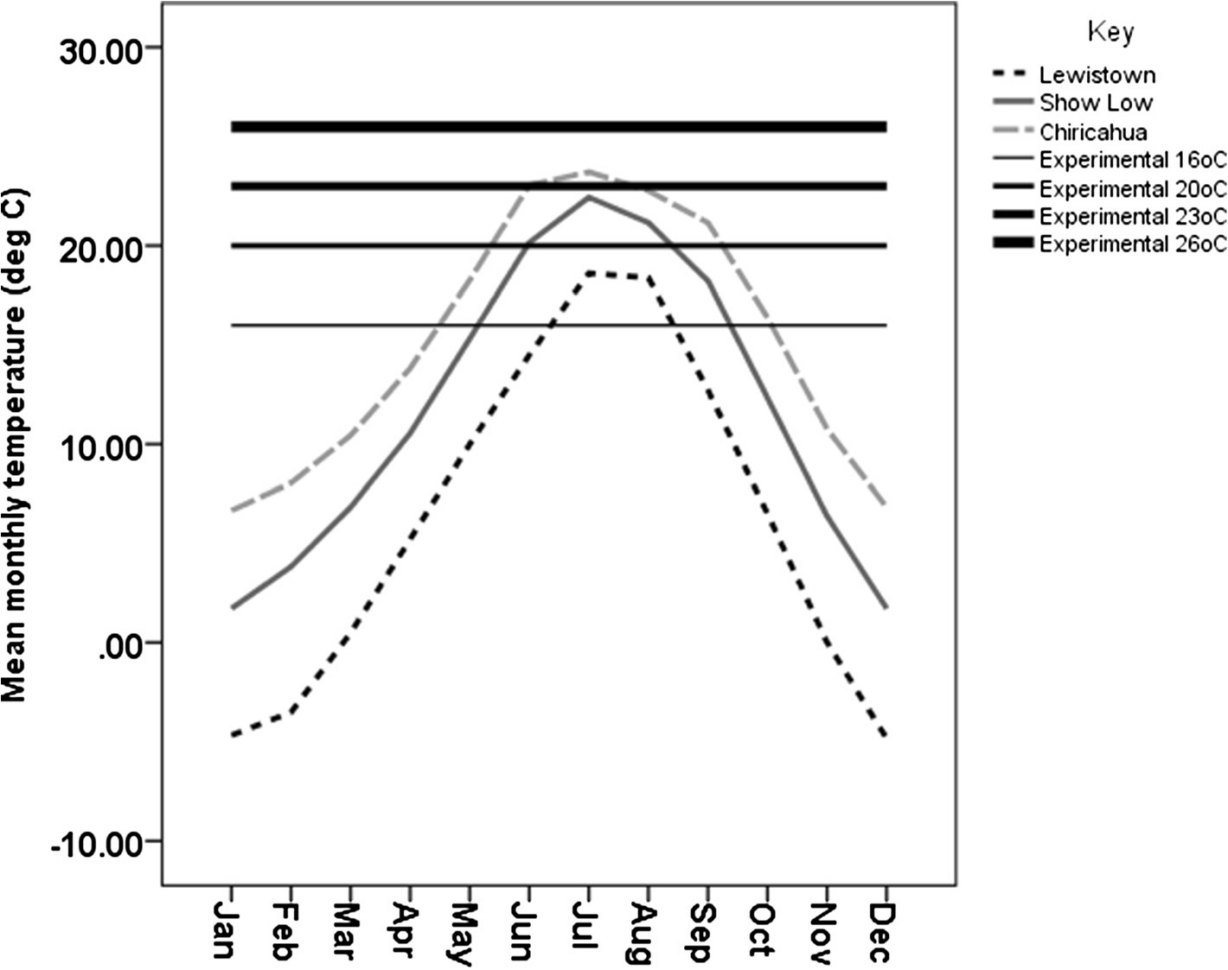
**Mean monthly temperature at each collection site.** Mean monthly temperatures in the three geographic locations relative to the experimental temperatures used in the experiment. Climate data from 1981–2010 is from the archives of the National Climatic Data Centre (National Oceanic and Atmospheric Administration).

Here we examine temperature-size relationships in *Drosophila pseudoobscura*. The geographic range of this fruit fly extends from Canada at the northern end of North America, through the USA into Guatemala (approximately 35 degrees of latitude, or 3800 km). Previous work has shown that phenotypic plasticity in body size occurs in this species [19]. Experimental work has also demonstrated the potential for evolutionary divergence between populations as plasticity in body sizes can stabilize over time under laboratory conditions [27,28]. Additionally, strong latitudinal distributions of other traits, such as sex ratio distortion and polyandry in this species have been observed but not yet conclusively explained [29]. One potential explanation is that phenotypic plasticity in body size, driven by temperature, underpins the latitudinal patterns already established in other traits. For example, larger females developing in cooler climates may produce substantially more offspring, requiring multiple matings to gain sufficient supplies of viable sperm for complete fertilization. Our aims are to investigate the variation in body size due to environmental, genetic, and genotype-by-environment (GxEs) influences. To achieve this, we examined 27 isolines representing three populations spanning a 1770 km latitudinal section of the geographic range across the USA. These populations experience an average of 5°C difference in mean monthly peak temperatures (Figure 1).

## Results

**Figure 2:**
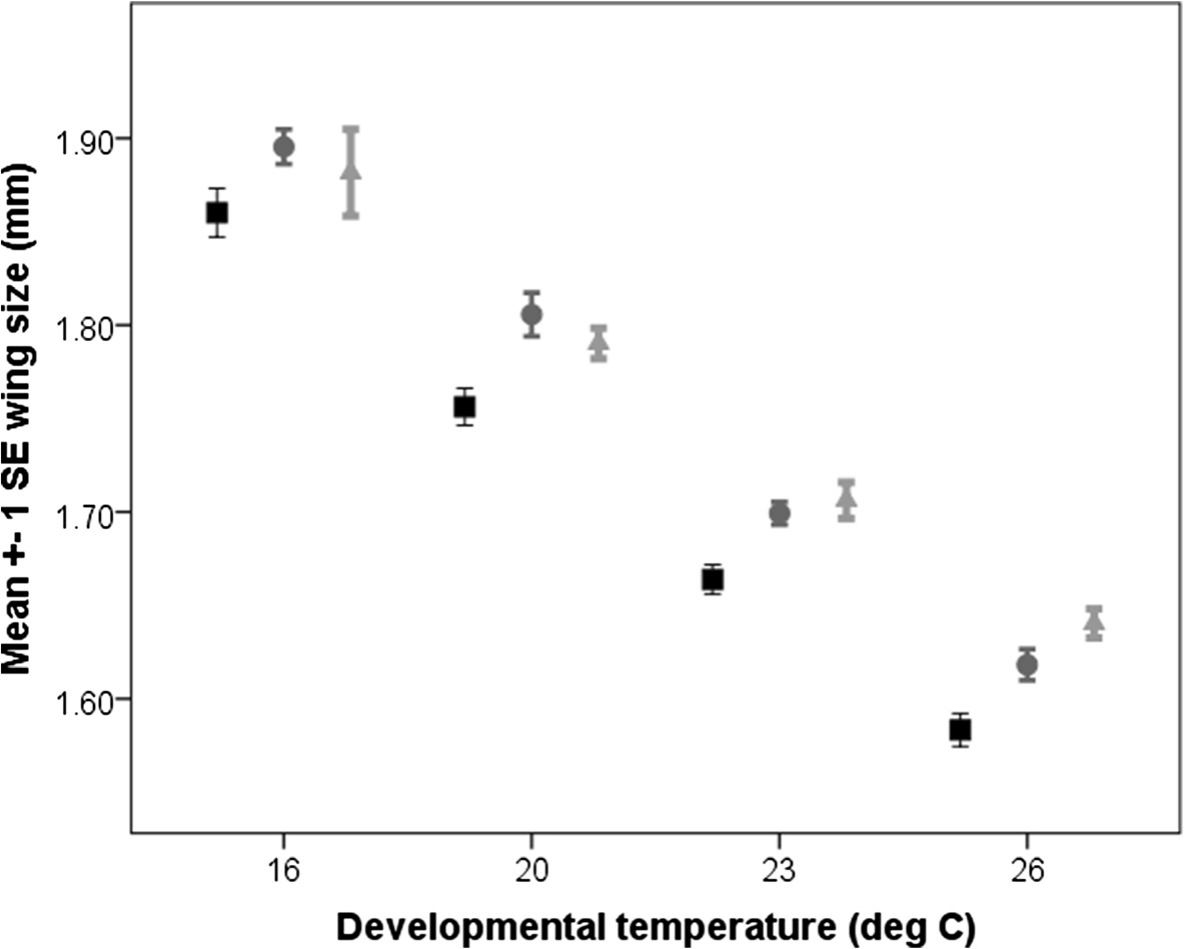
**Mean wing size of three populations at four temperatures.** Mean wing size (mm ± 1 se) scored across four developmental temperatures in three populations of flies. Lewistown (northern population) = black lines/square markers; Show Low (southern population) = grey lines/circle; Chiricahua (southernmost population) = grey lines/triangle markers.

We measured wing sizes in a total of 439 flies from 27 isolines across four temperatures. Summary statistics for wing sizes at different rearing temperatures and in different isolines are given in Table 1 and displayed in Figure 2. A full explanation of data analysis is included in the methods section. Briefly, wing length (WL) was scaled to standard deviation units prior to analysis and then modelled as: *WL* = *μ* + *Year* + *T* + *Pop* + *T. Pop* + *T. Year* + *ISO* + *T. ISO* + *ε*.

**Table 1:** **Summary statistics for wing sizes (mm) in 27 isolines of flies reared at four temperatures**

**Variable**	**N**	**Min**	**Max**	**Mean**	**SE**
**Temperature**	**Females**				
16°C	108	1.645	2.045	1.879	.007
20°C	116	1.538	2.025	1.782	.006
23°C	115	1.564	1.812	1.686	.004
26°C	100	1.473	1.739	1.607	.005
**Population**	**Isolines**				
Lewistown	9	1.473	2.024	1.718	.009
Show Low	12	1.482	2.045	1.755	.009
Chiricahua	6	1.589	1.998	1.757	.01

**Figure 3:**
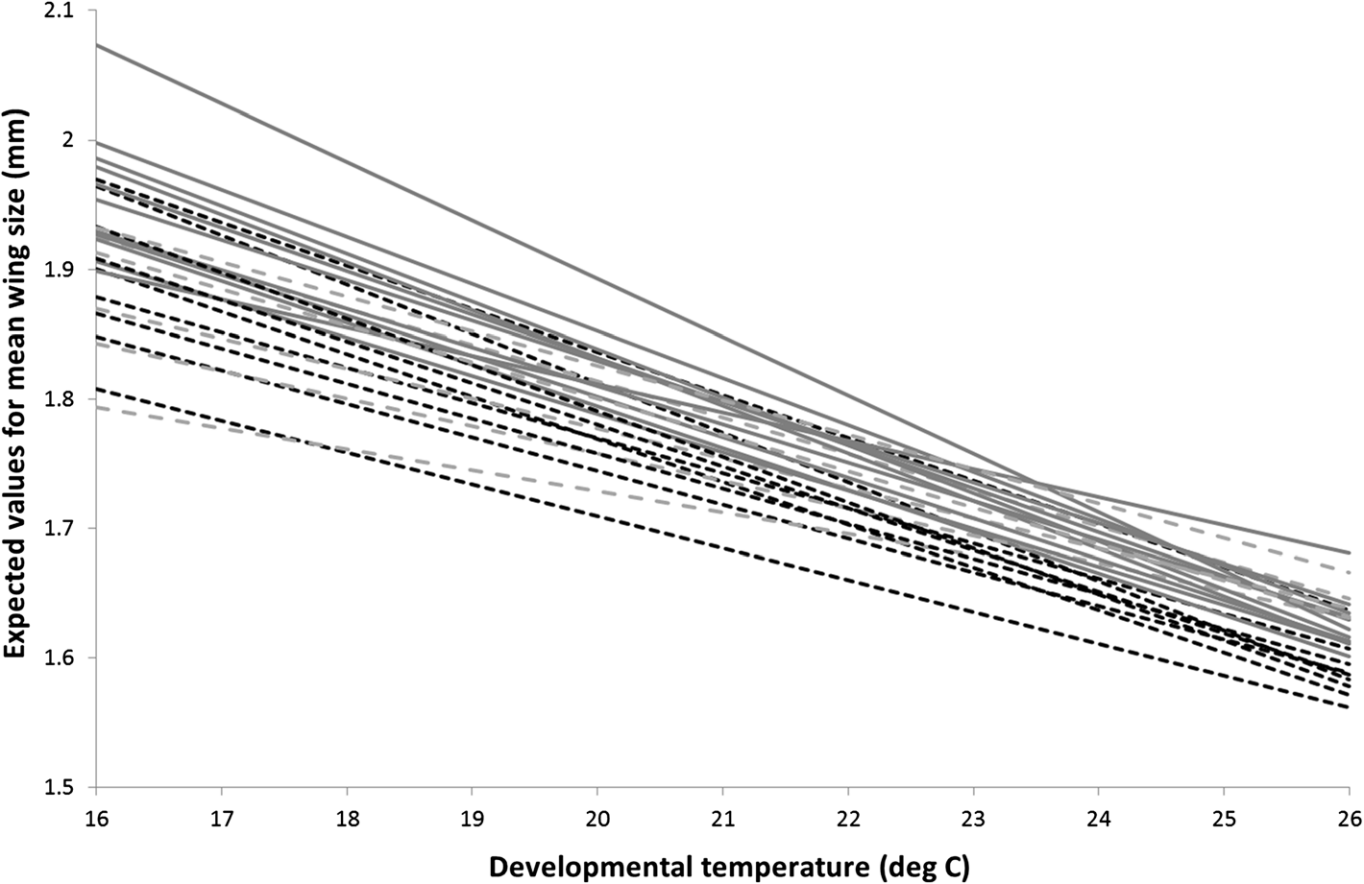
**Reaction norms for wing sizes (expected values) of each isoline, with temperature as a continuous variable.** Lewistown (northern population) = dashed black lines; Show Low (southern population) = solid grey lines; Chiricahua (southernmost population) = dashed grey lines.

Where, *μ* is the mean, *Year* is a 2-level fixed factor (2008, 2012), *T* is a linear effect of temperature in °C (mean centred across all observations), *Pop* is a 3-level fixed factor (Lewistown, Show Low, Chiricahua) and *Year.Pop* and *T.Year.Pop* are interaction terms. Our mixed model analysis showed that, after controlling for a small but significant Year effect and a marginally non-significant *T.Year* interaction effect, there was evidence for plasticity and among-population genetic variance in wing length (Table 2). Average wing length decreases at a rate of 0.201 (0.027) standard deviations units (approximately 0.024 mm) for each degree of temperature rise. However, there was no statistical support for among-population GxEs (represented in our model by the *T.pop* interaction term). Likelihood ratio tests provided evidence of significant within-population (among isoline) genetic variance (χ^2^_1_ = 81.2, P < 0.001) and GxE (χ^2^_1_ = 37.9, P < 0.001). Under the full model (i.e. including GxEs) the (co) variance parameters (SE) were estimated as: V_R_ = 0.153 (0.011), V_ISO_int_ = 0.064 (0.022), V_ISO_slp_ = 0.026 (0.001) and COV_ISO_int.slp_ = −0.007 (0.003), the latter scaling to an intercept-slope correlation r_ISO_int.slp_ of −0.571 (0.198). Note that since *T* was mean centred and the raw data scaled to unit variance, V_ISO_int_ can be directly interpreted as the proportion of observed phenotypic variance in wing length attributable to genetic differences among isolines at an average temperature (Figure 3). Overall we conclude that variation in wing sizes is primarily influenced by environmental temperature, with a smaller influence due to genetic variation expressed as significant isoline responses. There were no substantial influences on mean plasticity between populations due to GxE effects, but significant GxE isoline differences.

**Table 2:** **Fixed effect estimates from linear mixed effect model of wing length**

**Effect**	**Factor level**	**Coefficient**	**F**	**DF**	**P**
*μ*		14.64 (0.115)	73926	1,21.9	<0.001
*Year*	2012	0 (−)	6.35	1,20.6	0.020
	2008	−0.384 (0.122)			
*T*		−0.201 (0.027)	418	1, 20.7	<0.001
*Pop*	Chiricahua	0 (−)	5.57	2,21.9	0.011
	Lewistown	−0.178 (0.155)			
	Show Low	0.218 (0.156)			
*T.Pop*	Chiricahua	0 (−)	2.49	2,22	0.106
	Lewistown	−0.057 (0.035)			
	Show Low	−0.078 (0.035)			
*T.Year*	2012	0 (−)	4.09	1,19.4	0.057
	2008	0.052 (0.026)			

NB. The predicted mean (μ) is for a Chiricahua fly in 2012 at mean temperature (°C).

## Discussion

We examined temperature-size relationships in isolines of *D. pseudoobscura* collected across a latitudinal transect to establish the relative contributions of phenotypic plasticity and genotypic variation to variation in body size across the species geographic range. The strong relationship of body size with temperature can explain latitudinal patterns of species distributions via its close links with many aspects of fitness [21,23]. This can potentially also explain geographic patterns of variation in other traits [4,16]. Overwhelmingly, we found that environmental rearing temperature had the greatest effect on wing size. The substantial environmental influence of temperature on body size corroborates work in this and other *Drosophila* species that repeatedly show temperature-size relationships consistent with classic Bergmann clines – larger body sizes at lower temperatures [19-21,30]. Our absolute measures of wing sizes also closely resemble those measured using identical methods in other populations of *D. pseudoobscura*, suggesting that the overall range of phenotypic responses to specific temperatures has been well conserved over time in this species [27,28,31]. There are a multitude of theories to explain the enduring nature of temperature-size relationships, including metabolic rate and development rate [12-15]. The near-universal impact of temperature observed across all of the 27 genotypes suggests that the plastic response to temperature has remained constant across all three populations with little evolutionary divergence across the geographic range examined.

We also found evidence for genetic differences among isolines (and populations) in wing sizes. However, an entirely unexpected feature to emerge from our analysis was evidence consistent with a *converse* Bergmann cline, as flies from the northern population of Lewistown, which experience the coolest year-round climate, were genetically *smaller* than flies from the southern populations of Show Low and Chiricahua when reared at the same temperature. This is somewhat counter to evidence from Sokoloff, who found that body sizes of *D. pseudoobscura* derived from locations around the USA and Mexico did not show a clear relationship with latitude or local temperatures when reared at a standard temperature in the laboratory [32,33]. Opposing environmental and genetic variation in traits, known as countergradient variation and formally denoted by negative covariance between genetic and environmental sources of variance, can produce phenotypic similarity across different environments [22]. Given that we found significant phenotypic variation in body sizes across different environments, it is unlikely that countergradient variation alone is sufficient to explain patterns of body sizes in this case. Further, since we have data from only three populations from the potentially hundreds across the entire geographic distribution we refrain from making firm conclusions on this point. Interestingly, *D. pseudoobscura* tends to differ from the consensus pattern of adaptive distributions found in other traits in *Drosophila* species. For example, Kellermann *et al*. reported that global distributions of 30 tropical and cosmopolitan *Drosophila* species could be explained by genetic variation in cold-stress with tropical species effectively confined to their lower latitudes by lack of suitable genetic variation in their ability to tolerate cold-stress [26]. In contrast, *D. pseudoobscura* from colder climates are equally as tolerant of hot conditions as those originating from warmer climates, and those from warmer climates are better at tolerating cold shocks than those from colder sites [25].

One possibility is that the unique system of third chromosome inversions in this species has influenced the genetic diversity available within populations that enables them to respond effectively to environmental conditions [34]. In our populations, individuals heterozygous for inversion sequences are more common in our northern laboratory populations, which may have preserved small body sizes in this population [35]. However, we note that the closely related *D. subobscura* also has chromosome inversions and yet shows the more common pattern of larger body sizes at lower temperatures due to selection for efficient wing loading at different body sizes [17,18]. It is possible that by using a selection of isolines that were collected in 2008 and 2012 we have inadvertently measured laboratory adaptation in wing sizes and interpreted this as genetic differences between populations. However, we do not think this is likely as the purpose of inbreeding from isofemale lines immediately after capture from the field is to prevent laboratory adaptation and preserve the genetic variation available [36], but see [37,38] for cases where laboratory adaptation occurred in isofemale lines. We also specifically accounted for the effect of ‘year’ in our analyses and found no evidence that year of collection is driving the effects observed. Alternatively, body size may be under indirect selection by being closely linked with other fitness related traits, which may themselves have no relationship with temperature [39]. For example, the relationship between body size and mating success or fecundity may differ in northern and southern populations of *D. pseudoobscura*, which may prevent convergence between environmental and genetic determination of body sizes.

We also found significant genetic variation in plasticity (GxEs) between isolines within, but not between, populations. In other words, looking among populations there is no more GxE than expected given each population itself was comprised of a unique mixture of isolines. Thus there is no evidence that *mean* plasticity has diverged among populations due to local selection. Interestingly, we observed from the reaction norms that the isolines in our experiment deviated most from each other at the coldest temperature (Figure 3), suggesting that genotypes began to show differences in the ability to grow and develop at this temperature. This indicates that some genotypes may be more susceptible to cold stress than others, but that cold-tolerance does not appear to differ between populations.

The relative contributions of phenotypic plasticity (environmental variance and GxEs) and genetic variance to body size variation closely resemble work by Gupta & Lewontin, who compared the number of thoracic bristles in natural populations of *D. pseudoobscura* reared at 14°C, 21°C and 26°C [31]. They also compared reaction norms and found that all individual genotypes conformed to a narrow range close to the average response across all temperatures, suggesting that a range of morphological traits in *D. pseudoobscura* are liable to show plastic responses to temperature and with only limited influence of GxEs. GxEs have been posited as a key component to the maintenance of genetic variation [8]. So, while it is possible that small, but significant, GxE effects could promote or disrupt adaptation to different environments, in this case, the influence of environmental temperature over any genetic effects suggests that these populations of *D. pseudoobscura* have not significantly diverged into separate ‘specialist’ populations.

## Conclusions

Temperature is the strongest influence on body sizes in *D. pseudoobscura* and can result in significant differences in adult flies inhabiting different climates with flies reared in cooler temperatures being significantly larger. However, there is also significant genetic variation for body size at both among- and within-population levels. Among populations, genetic effects on body size run counter to the environmental influence as genotypes from the southern populations grew larger at all temperatures than those from the northern population. This could be driven by unique systems of chromosome inversions, or due to indirect selection on body size via correlated traits. Genetic variance in plasticity is present within populations, but there is no evidence of local adaptation in plasticity itself.

## Methods

### Origin of the flies used

In 2008 and 2012 we collected wild *D. pseudoobscura* from three locations in North America. We used banana baits to collect flies from Lewistown, Montana (47°03′ N, 109°28′ W), Show Low, Arizona (34°16′ N, 110°00′ W) and Chiricahua, Arizona (31°54′ N, 109°15′ W). We completed collections during the late spring season when populations are highly abundant and used individual wild-caught females to establish isofemale lines. Isofemale lines are created by inbreeding the offspring of a single wild-caught female, which rapidly become homozygous at most alleles, making individuals within an isoline virtually genetically identical, and minimizing adaptation to laboratory conditions [36] (but see [37,38]). Hence they maintain their wild genotype, and a set of isolines maintains natural genetic diversity far more effectively than a mass laboratory population that would evolve in laboratory culture [36]. We also used isolines in preference to alternative quantitative genetic approaches to minimize missing data and effectively implement our treatment combinations of temperature and genotype [40]. We initially maintained all flies at 23°C on a 14:10 light: dark cycle and cultured them on 8 ml of standard *Drosophila* porridge medium containing water, oats, sugar, brewer’s yeast, and agar, plus a dilute solution of nipagin (2 g/litre of porridge medium, dissolved in 10 ml of ethanol) and propionic acid to inhibit mould and bacteria growth.

We allowed several generations of inbreeding (c. 40 generations for flies collected in 2008, 4 generations for flies collected in 2012) to account for carry over effects from the field and drift within the lab. We then selected 27 isolines for the experiment (9 from Lewistown (4 from 2008, 5 from 2012), 12 from Show Low (6 from 2008, 6 from 2012), and 6 from Chiricahua (all 2012)). Some southern USA populations of *D. pseudoobscura* harbour a meiotic driving X chromosome that can impact on temperature sensitivity [41]. We checked that none of our isolines carried this chromosome by screening them with a PCR assay, and by checking for all female broods produced by males from the isolines [42].

### Experimental temperatures

To calibrate our environmental conditions we collected long-term climatic data from the archives of the National Climatic Data Centre at the National Oceanic and Atmospheric Administration [43]. We used mean monthly temperatures compiled over the last thirty years (1981–2010) to construct annual temperature ranges for the three geographic locations of *D. pseudoobscura* in our experiment (Figure 1). From this figure we chose a range of developmental temperatures that would allow us to examine plasticity in both the natural range experienced by the flies in our experiment and at temperatures beyond the natural range experienced by these populations of *D. pseudoobscura* to quantify the extent of plasticity in body size. We chose average monthly temperatures as we were interested in developmental plasticity during the larval stage, when individuals spend the majority of their time within a food substrate and are likely to be less exposed to the extremes of daily temperatures.

### Assay of body size at a range of temperatures

To examine genetic variation in plasticity of body size due to temperature during development we split each isoline into groups and reared them at different developmental temperatures: 16°C, 20°C, 23°C and 26°C. For each isoline at each temperature we set up five vials, each containing two males and two females. We then used the offspring from these flies to initiate experimental vials, where three males and three females per vial were allowed to mate and lay eggs for seven days. By standardising the number of flies per vial, and the laying time, we ensured excess food per larvae in each vial, avoiding any size differences due to larval competition. Thus our experimental flies were cultured in their respective temperature treatments for two generations prior to measurement to avoid any maternal or lag effects from the standard 23°C culture.

As a proxy for overall body size we measured wing length as this trait has been shown to correlate with other measures of body size and is easily standardized between individuals [27,44]. We restricted our analysis to female flies as female body size is most closely associated with fecundity and population fitness, and previous work has established that there is no sexual differentiation in phenotypic plasticity in wing sizes in this species [27,28]. To measure wing sizes, we froze all of the virgin females that eclosed from each vial at four days of age and then selected 5 females at random to represent each isoline/temperature treatment. We then cut off the wings at the humero-costal break (shoulder) and mounted them on a slide with 1 drop of Hoyer’s solution and secured them with a cover slip. We photographed all wings at ×32 magnification using a Leica dissecting microscope attached to a Leica DFC295 digital camera. We then measured wing lengths on the digital image using ImageJ software and applying a calibration of 600 pixels: 1000um [45]. Wing length was measured as the straight line distance from the junction of the 3^rd^ longitudinal vein and the anterior cross vein to the distal wing margin of the 3^rd^ longitudinal vein. For each female we measured both left and right wings and then calculated mean wing length in millimetres. Further information on our study species can be found at the ‘Wedell Polyandry Website’ [46].

### Data analysis

Wing length data for each isoline conformed well to a normal distribution. We used linear mixed effect models fitted by restricted maximum likelihood (REML) using the program ASREML to test the effects of genotype, environment (temperature), GxE on wing size [47]. Note that genetic variance (and GxE) can be present both among- and within-populations in our study. Under our common garden design the former would be evidenced by significant variation in wing length among-populations, the latter by variation among-isolines (within populations). Wing length (*WL*) was scaled to standard deviation units prior to analysis and then modelled as:

$$ WL = \mu + Year + T + Pop + T. Pop + T. Year + ISO + T.ISO + \varepsilon $$

Where, *μ* is the mean*, Year* is a 2-level fixed factor (2008, 2012), *T* is a linear effect of temperature in °C (mean centred across all observations), and *Pop* is a 3-level fixed factor (Lewistown, Show Low, Chiricahua). Interaction terms of *Year.Pop* and *T.Year.Pop* were initially tested but being non-significant and not directly relevant to hypotheses being tested were not included in models shown here. We then included random intercept (*ISO*) and slope (*T.ISO*) terms to model within-population (among-isoline) genetic variance and GxE. Random slope and intercept terms were assumed to be normally distributed with mean of zero and a covariance structure to be estimated. This covariance structure contains the estimates of variance among isoline intercepts (V_ISO_int_) and slopes (V_ISO_slp_), and the among-isoline intercept-slope covariance (COV_ISO_int.slp_). Residual errors (*ε*) are assumed to be normally distributed (with mean zero and variance V_R_) and uncorrelated across observations. We assessed the significance of fixed effects using conditional F-tests implemented in ASREML, and employed likelihood ratio tests for statistical inference on random effects. Specifically, to determine the significance of within-population GxE we compared the full model to one in which no *T.ISO* term was fitted. We then dropped the *ISO* term as well to test the significance of within-population genetic variance. We generated norms of reaction for all isolines using expected values from this model (Figure 3).

## Availability of supporting data

The data sets supporting the results of this article are included within the article (and its additional files).

## Additional file

Additional file 1:
**Raw data for wing sizes of female** ***D. pseudoobscura*** **collected from different populations across North America and reared at different experimental tempeatures.**
